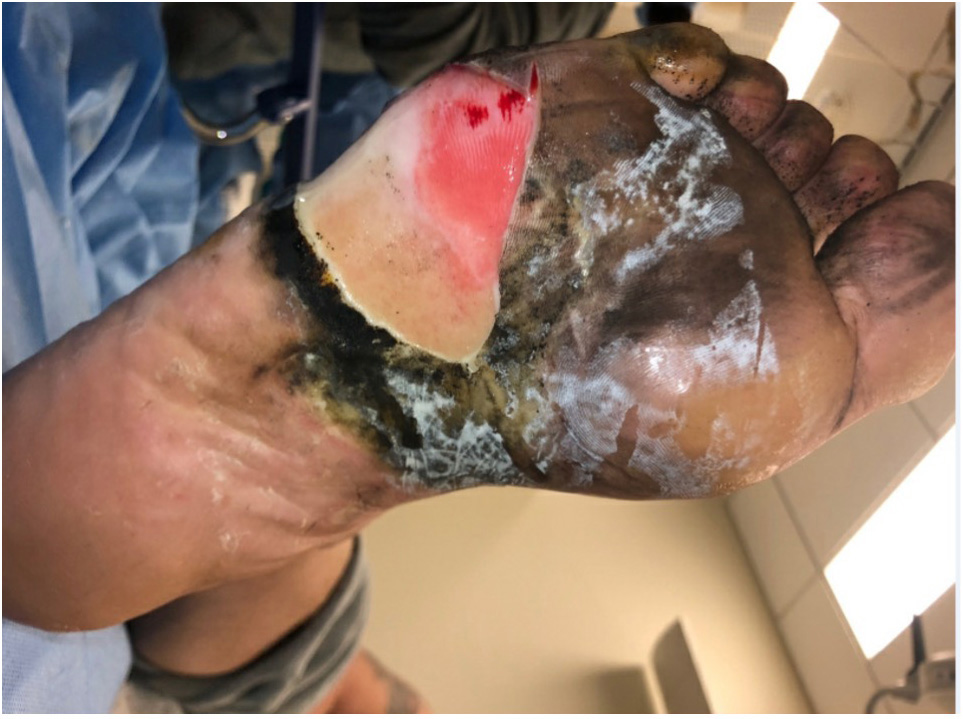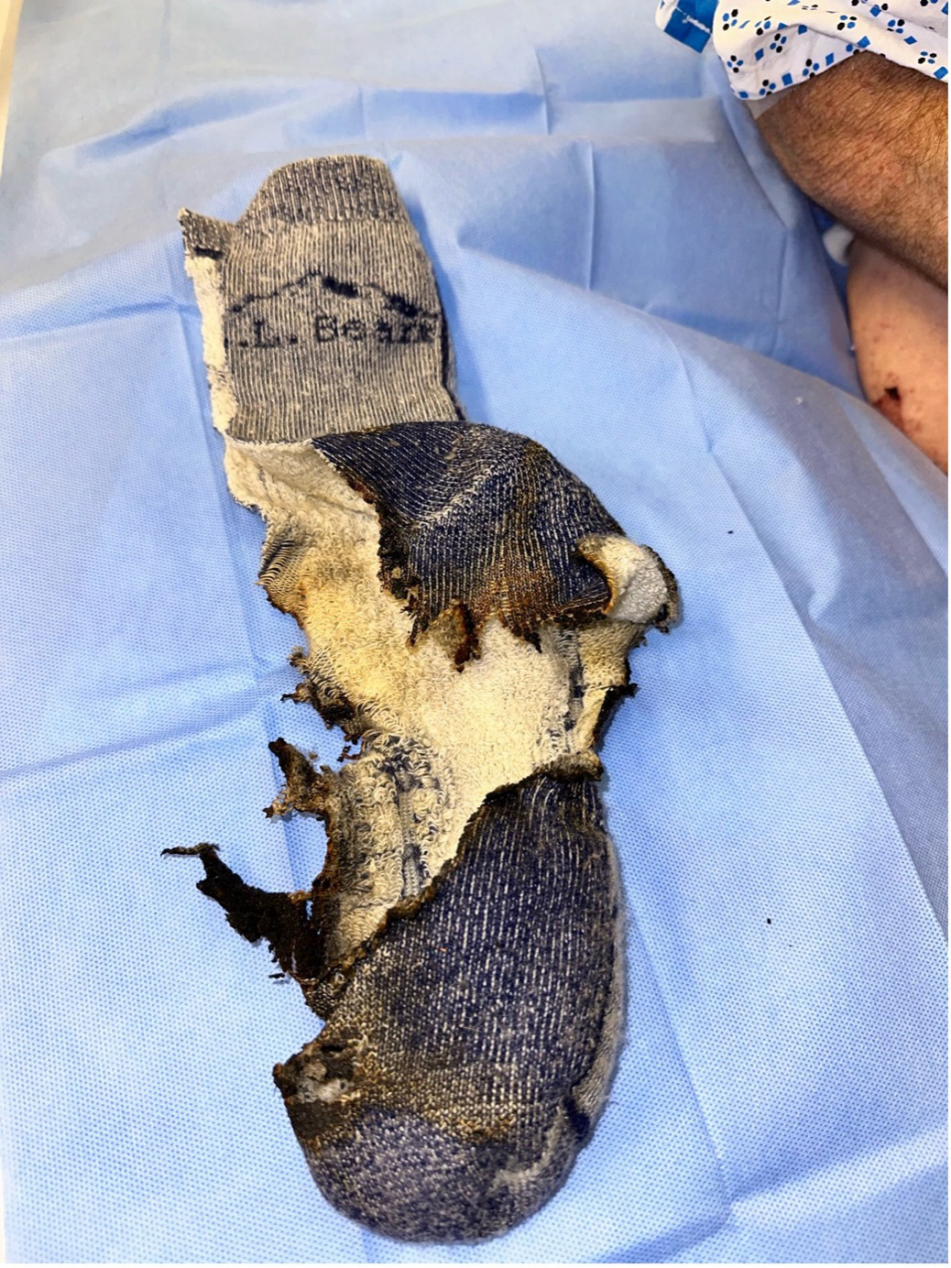# 647 Full Thickness Burn Injuries from Heated Insole Device Explosions: First Case Series

**DOI:** 10.1093/jbcr/iraf019.276

**Published:** 2025-04-01

**Authors:** Harvak Hajebian, Courthney chambers, Dinora Murota, Rachelle Lodescar, Joseph Turkowski

**Affiliations:** Garnet Health Medical Center; Westchester Medical Center; New York Medical College; Burn Center at Westchester Medical Center; Westchester Medical Center

## Abstract

**Introduction:**

Lithium-ion batteries, developed in the 1990s, are commonly used in devices such as laptops, smartphones, and medical equipment. One application is heated insoles, designed to warm feet during activities like hiking and skiing. These insoles have thin heating elements between layers of insulation, powered by a rechargeable battery typically in the heel, reaching temperatures of 110 to 140 degrees Fahrenheit for up to 8 hours. They feature adjustable heat settings and automatic shut-off for safety. However, overheating of the battery’s electrolyte can lead to “thermal runaway,” causing cell rupture or combustion. While explosions have been documented in other devices, there are no reported burn injuries from heated insoles.

**Methods:**

A single-institution review of the clinical presentation, management, and proposed treatment algorithms for three separate cases of full-thickness burn injuries caused by spontaneously exploding heated insole devices was performed at an American Burn Association verified burn center during a wintertime three-month span between December 2023 to February 2024.

**Results:**

Three cases highlight burn injuries from lithium battery-powered heated insole devices (HIDs). A 40-year-old male suffered 1.5% TBSA burns to his right foot and ankle after an HID ignited while operating a construction vehicle; he underwent skin grafting and recovered well. The second case involved a 48-year-old male who sustained a 0.5% TBSA burn to his right heel while using a lawn mower, leading to tangential excision and successful skin grafting. The third case was a 75-year-old male who experienced a 1.5% TBSA burn from an ignited HID in his snow boots and required excision and skin grafting, achieving mostly satisfactory healing.

**Conclusions:**

This report presents three cases of burn injuries from heated insole device explosions that required surgical skin grafting. These injuries can involve both thermal and chemical damage to the foot and ankle, necessitating careful evaluation for potential metallic toxicity. First aid for exposure to the contents of lithium-ion batteries includes a thorough irrigation of the affected site with water. Rechargeable lithium-ion batteries do not contain lithium metal, thus the use of water is safe and appropriate. There is currently no consensus on treatment protocols for these burns. Mandatory product labels outlining proper use and associated risks, along with improved regulation of lithium-ion devices, may be essential to reduce the incidence of explosion-related burn injuries.

**Applicability of Research to Practice:**

As the incidence of exploding lithium-ion powered devices continues to rise, burns resulting from heated insole device explosions may become a more prevalent presentation in the acute care setting. For acute care physicians, the recognition of these injuries is paramount in initiating an appropriate management algorithm, which often includes referral to a regional burn center.

**Funding for the Study:**

N/A